# An interactive patient transfer network and model visualization tool for multidrug-resistant organism prevention strategies

**DOI:** 10.1017/ash.2023.403

**Published:** 2023-09-29

**Authors:** Rany Octaria, Samuel Cincotta, Jessica Healy, Camden Gowler, Prabasaj Paul, Maroya Walters, Rachel Slayton

## Abstract

**Background:** The CDC’s new Public Health Strategies to Prevent the Spread of Novel and Targeted Multidrug-Resistant Organisms (MDROs) were informed by mathematical models that assessed the impact of implementing preventive strategies directed at a subset of healthcare facilities characterized as influential or highly connected based on their predicted role in the regional spread of MDROs. We developed an interactive tool to communicate mathematical modeling results and visualize the regional patient transfer network for public health departments and healthcare facilities to assist in planning and implementing prevention strategies. **Methods:** An interactive RShiny application is currently hosted in the CDC network and is accessible to external partners through the Secure Access Management Services (SAMS). Patient transfer volumes (direct and indirect, that is, with up to 30 days in the community between admissions) were estimated from the CMS fee-for-service claims data from 2019. The spread of a carbapenem-resistant Enterobacterales (CRE)–like MDROs within a US state was simulated using a deterministic model with susceptible and infectious compartments in the community and healthcare facilities interconnected through patient transfers. Individuals determined to be infectious through admission screening, point-prevalence surveys (PPSs), or notified from interfacility communication were assigned lower transmissibility if enhanced infection prevention and control practices were in place at a facility. **Results:** The application consists of 4 interactive tabs. Users can visualize the statewide patient-sharing network for any US state and select territories in the first tab (Fig. 1). A feature allows users to highlight a facility of interest and display downstream or upstream facilities that received or sent transfers from the facility of interest, respectively. A second tab lists influential facilities to aid in prioritizing screening and prevention activities. A third tab lists all facilities in the state in descending order of their dispersal rate (ie, the rate at which patients are shared downstream to other facilities), which can help identify highly connected facilities. In the fourth tab, an interactive graph displays the predicted reduction of MDRO prevalence given a range of intervention scenarios (Fig. 2). **Conclusions:** Our RShiny application, which can be accessed by public health partners, can assist healthcare facilities and public health departments in planning and tailoring MDRO prevention activity bundles.

**Figure:**
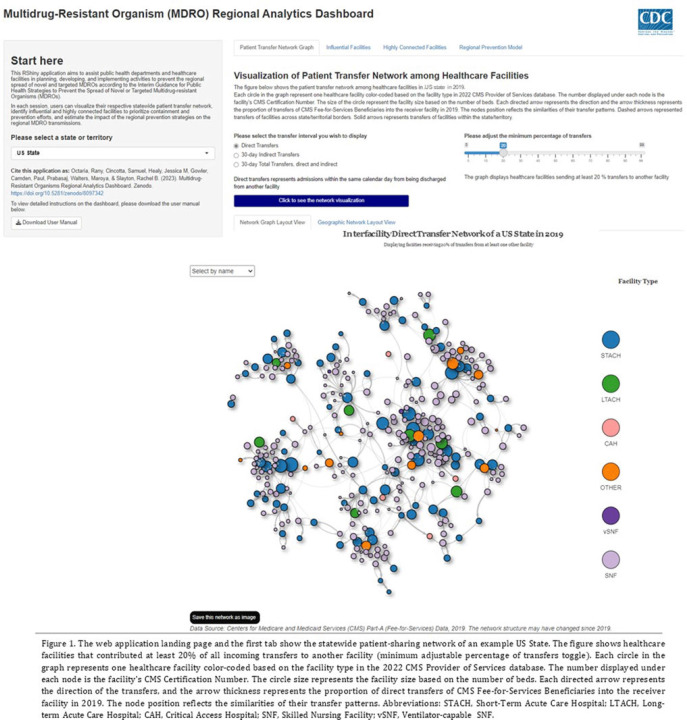

**Figure:**
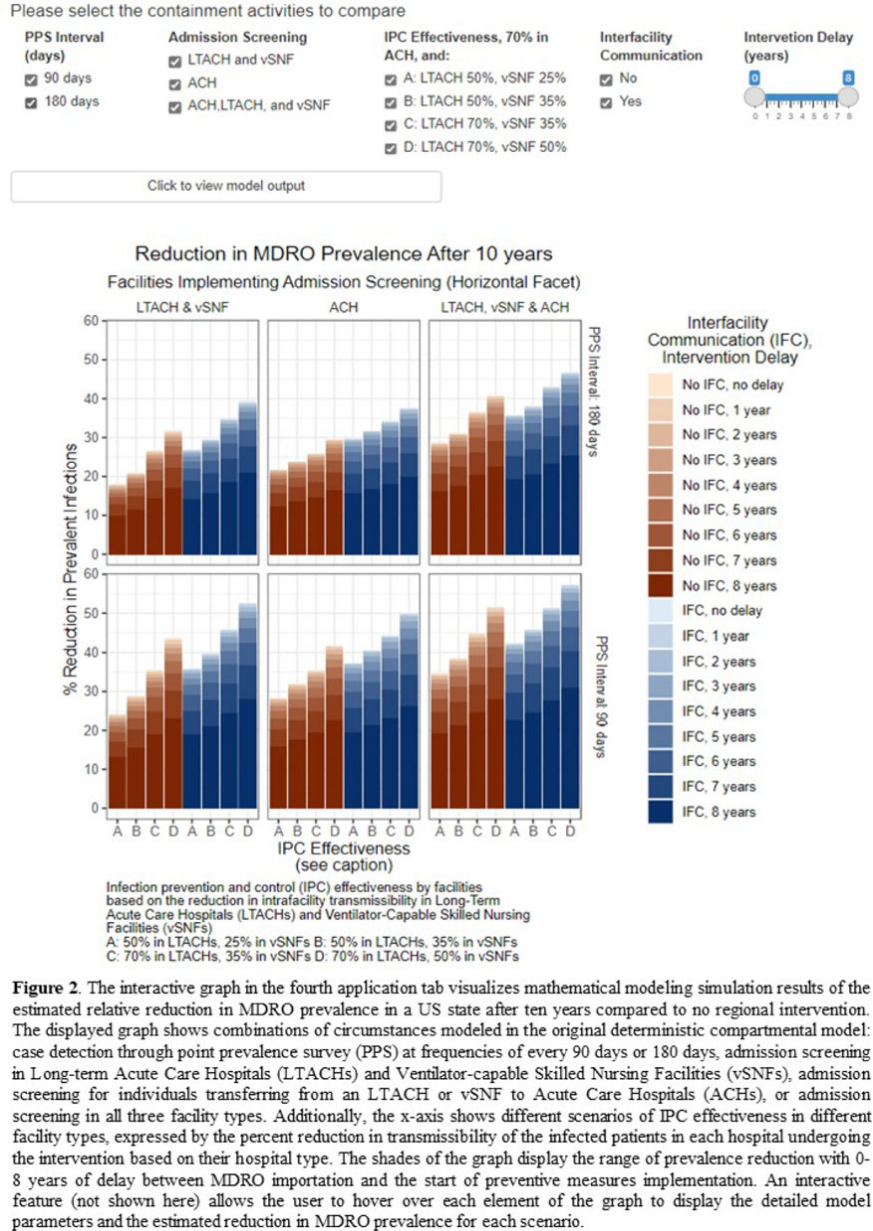

**Disclosures:** None

